# Remote sensing assessment of the weed adaptability to soil salinization induced by extreme droughts on coastal agriculture

**DOI:** 10.1016/j.isci.2025.112410

**Published:** 2025-04-11

**Authors:** Nebojša Nikolić, Sara Cucchiaro, Eugenio Straffelini, Paolo Tarolli, Roberta Masin

**Affiliations:** 1 Department of Agronomy, Food, Natural resources, Animals and Environment, University of Padua, Legnaro, PD 35020, Italy; 2 Department of Agricultural, Food, Environmental and Animal Sciences, University of Udine, Udine, UD 33100, Italy; 3 Department of Land, Environment, Agriculture and Forestry, University of Padua, Legnaro, PD 35020, Italy

**Keywords:** Earth sciences, Environmental science, Remote sensing, Plants, Agricultural science

## Abstract

Salinization and drought pose significant challenges to agriculture in coastal regions, yet their combined impact on crop production and weed proliferation remains understudied. This study investigated the influence of salinity caused by extreme droughts on agricultural ecosystems in the Po River Delta (Italy), using remote sensing techniques and soil measurement, focusing on crop health and weed resilience. Our findings reveal that prolonged drought conditions are exacerbated by saline water intrusion and elevated soil salinity levels, particularly in fields closer to the coast. While crops, notably soybeans, exhibited susceptibility to salinity stress, weeds displayed remarkable resilience, thriving in adverse conditions and outcompeting crops. Notably, weed populations showed increased density and adaptability, even in areas of high salinity and drought. These findings underscore the urgent need for comprehensive strategies to mitigate the impact of salinity and drought on crop productivity and manage weed infestations in coastal agricultural areas.

## Highlights

• Extreme drought-driven salinity boosts weed dominance over crops
• UAV monitoring reveals weeds thriving where crops fail under salinity stress
• Coastal agriculture faces rising weed threats from climate-driven salinization
• Results highlight urgent need for new weed control in hostile environments

## Graphical abstract

**Figure:**
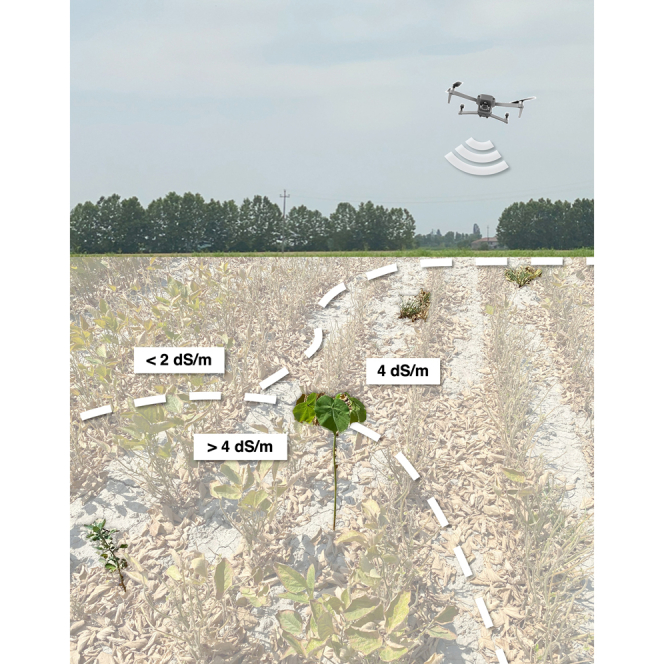

## Introduction

Soil salinization is a problem across Europe and is projected to worsen due to climate change, leading to an expansion of salt-affected soils.^1^^,^^2^^,^^3^ These soils, characterized by high concentrations of soluble salts, present significant challenges to crop growth and productivity.^4^^,^^5^ Furthermore, the negative effects of salinity are exacerbated by increasing temperatures.^6^ They typically have an electrical conductivity (EC) above 4 dS/m, but it is advisable to lower the threshold to 2 dS/m to mitigate negative outcomes for different plant species.^7^^,^^8^^,^^9^ Saline-affected soils currently occupy about 20–30% of total arable lands, a percentage expected to increase due to factors like low precipitation, high surface evaporation, irrigation with saline water, weathering of native rocks, and poor agricultural practices.^10^^,^^11^ Soil salinization is closely linked to climate change, where high temperatures and low precipitation can significantly increase salinity, especially in the root zone.^12^^,^^13^^,^^14^ This accumulation of salts in the root zone negatively impacts vegetation, particularly crop species.^15^^,^^16^^,^^17^ Furthermore, the risk of soil salinization, especially in the root zone, is heightened in croplands due to water evaporation and plant processes like transpiration, which can result in the release of freshwater that quickly evaporates, leaving behind salts that further increase soil salinity.^13^^,^^14^^,^^18^^,^^19^^,^^20^ One of the most at-risk parts of agricultural systems are surely the coastal areas. Agriculture in coastal areas is present around the world as these areas are usually highly suitable for agricultural production; they are relatively flat, fertile soils with usually good supply of water, from surface and/or subsurface sources, with a milder and more humid climate, which may favor growth of crops more than in other areas. Additionally, these areas offer different socioeconomic benefits which certainly contribute to their urbanization and the development of agriculture.^21^ In different European regions, the percentage of coastal areas used for agricultural production can reach up to 73%, while in the Veneto region, this percentage is estimated to reach 59%.^22^ In this region, one of the most characteristic areas of coastal agriculture is the one constituted by the Po River Delta which, after a series of land reclamation interventions, is now one of the major production areas, with waste extensions of grain, maize, soybean, and rice production fields.^23^^,^^24^ Coastal agriculture areas are projected to be increasingly at risk throughout this century and beyond due to global climate change. A major risk for these areas is the immediate exposure to the effects of the rise of the sea level and seawater intrusion, increasing the salinity of the soil.^25^ These phenomena can have an extremely negative impact on agricultural production in coastal areas, especially considering that agricultural production in these areas consists mainly of farming systems that are not specific to the coast but have developed in these areas due to farmers’ interests, as can be seen in the example of the Po River Delta.^24^^,^^26^ This means that coastal agricultural practices are far less stable than inland agricultural practices as they need to cope with frequent changes in salinity and water availability. This is particularly exacerbated in crop systems with low salinity tolerance, putting the food security in coastal zones^25^ at risk. However, abiotic stressors are not the only problem for coastal agriculture as there are also biotic stressors such as weeds, which may represent a significant challenge due to their competitive nature compared to crop species, and their financial impact on agricultural production is estimated to reach millions of euros worldwide.^27^^,^^28^^,^^29^^,^^30^^,^^31^^,^^32^ Different plant species exhibit varying responses to soil salinization, showing different levels of tolerance and adaptability on structural, physiological, and molecular levels.^33^^,^^34^^,^^35^^,^^36^^,^^37^^,^^38^^,^^39^ Interestingly, weed species are generally more resilient to high salinity levels and more adaptable to salinized agroecosystems than crop species.^40^^,^^41^^,^^42^ Therefore, as the climate changes, weeds are likely to pose an even more significant threat to agricultural production, as highlighted in some recent works.^43^ With the projected increase in global population to 8–10 billion by 2050 and 9.6–12.3 billion by 2100, coupled with the loss of arable lands due to various geological processes, climate change, and human settlement expansion, it becomes imperative to improve agricultural production and protect crops from adversities such as weeds.^44^^,^^45^^,^^46^^,^^47^ This need is further compounded in areas already affected by negative abiotic factors such as soil salinization, which is prevalent in coastal areas.^48^^,^^49^ Given the detrimental effects of weed competition on agricultural production, studying weed presence and distribution in agricultural areas affected by high soil salinity, such as coastal agricultural areas, is crucial. One potential approach to address this task is the utilization of innovative remote sensing technologies. Advancements in remote sensing, such as digital photogrammetry (e.g., structure from motion – SfM technique) coupled with unmanned aerial vehicles (UAVs), offer promising opportunities for weed mapping and in-field management.^50^^,^^51^^,^^52^^,^^53^^,^^54^ UAV surveys combined with geographic information systems (GIS) provide comprehensive assessments of weed spatial patterns and distribution, as weeds often form distinct patches.^55^^,^^56^ Integration of optical sensors, such as RGB, multispectral, or hyperspectral sensors, allows the acquisition of high-resolution images, facilitating accurate identification of weeds, even at early growth stages.^57^^,^^58^ Additionally, these techniques enable the monitoring of vegetation stress levels, including the influence of abiotic stresses like salinity.^59^^,^^60^^,^^61^ UAVs are also commonly used for mapping weeds and monitoring crop health. However, research applying these techniques in areas of high salinity or those at risk of salinization is still lacking. Previous studies have investigated the salinization of the coastal agricultural area of the Po River Delta, but these were conducted at low resolution using satellite images and did not focus on weed presence.^62^ To address this research gap, in this study, a global navigation satellite system (GNSS) and UAVs equipped with visible and multispectral sensors were employed to monitor and identify weed species in three different fields located in the Po River Delta, Northeastern Italy. The investigations were conducted in the summer of 2022, during one of the most severe droughts ever recorded in northern Italy that resulted in major saltwater intrusion into the delta with heavy damage to agriculture.^63^ Although a negative trend in precipitation was detected in this area, with a 31% precipitation reduction compared to the previous decade,^64^ the events of 2022 are described as unprecedented drought phenomena.^65^ Indeed, the data relative to 2022 for this area indicate a severe drought condition from June until half of August. These events coupled with the specific conditions and position of the Po River Delta caused a major saltwater intrusion of more than 40 km inland, adding additional stress on agricultural production in the area.^63^^,^^64^ Official estimates indicate that this event caused a 10% production loss in the Italian agricultural sector, translating to 6 billion euros of economic damage.^66^ Therefore, in addition to weed detection, the health of crop species was also monitored using the same technique. Salinity levels were determined by collecting soil samples from the root zone, and the results were interpolated to create salinity maps. These maps were then overlaid with weed distribution maps and crop health maps to assess the adaptability of weed species to varying soil salinity levels.

## Results

### Climatic conditions in the study area

**Figure 1:**
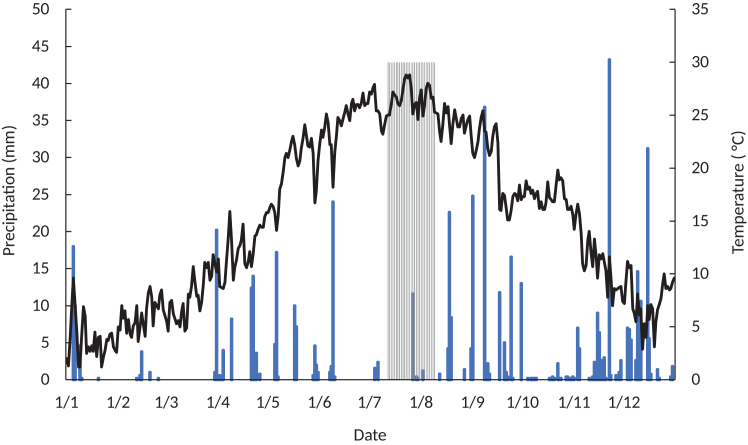
Precipitation and mean temperature in 2022 at Porto Tolle, the gray area indicates the period between the first (12/07/2022) and the second survey (09/08/2022)

Precipitation and temperature registered in 2022 are displayed in Figure 1. The Po River Delta suffered a significant decrease in precipitation and an increase in temperature compared to the historical levels as evidenced in the work of Straffelini et al.^64^ The month of July, for example, experienced a −65% rainfall anomaly compared to the long-term average, which contributed to the severe saltwater intrusion process.^64^

**Figure 2:**
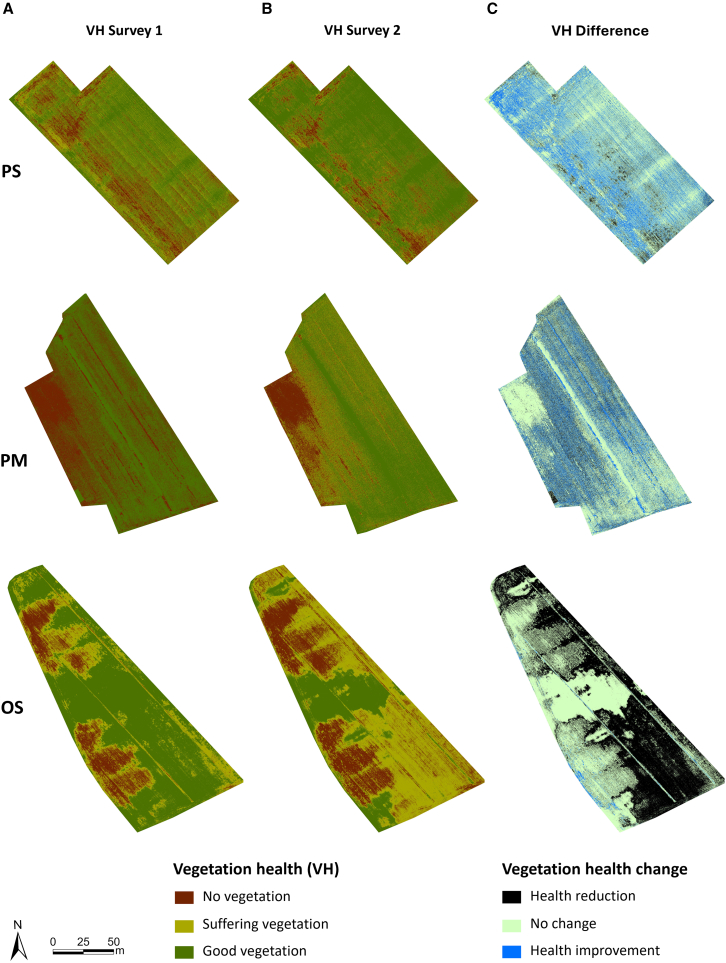
Vegetation health and changes in the experimental fields Vegetation health (VH), and Vegetation health change (VHC) in the experimental fields: (A) VH1 – first survey on 12 July 2022, (B) VH2 – second survey on 9 August 2022, (C) VHC – Vegetation health change from the first to the second survey PS- Polesinello soybean, PM Polesinello maize, OS- Oca Marina soybean.

As evidenced by the gray color in Figure 1, some precipitation events occurred between the first (July 12, 2022) and the second survey (August 9, 2022), with observable effects on the soil salinity levels of the fields and consequentially also on plant health (Figure 2).

### Variations in salinity across fields and the impact on vegetation

The salinity levels in the Polesinello soybean field remained consistently very low (≤2 dS/m) both during the first (July 12, 2022) and the second survey (August 9, 2022). Despite the low salinity levels recorded during the initial and subsequent surveys, signs of vegetation stress were evident in certain areas of the field (Figure 2). Additional details can be found in Table 1.

**Table 1:** The area of VH levels measured during the first and the second survey and the DoVH between the two surveys

Vegetation health	First survey (m^2^)	Second survey (m^2^)	Health change	Area (m^2^)
No vegetation	3616.5	2339.67	Reduction	1286.31
Suffering vegetation	5627.13	3217.43	No change	11237.94
Good vegetation	8767.21	12453.74	Improvement	5486.58

**Figure 3:**
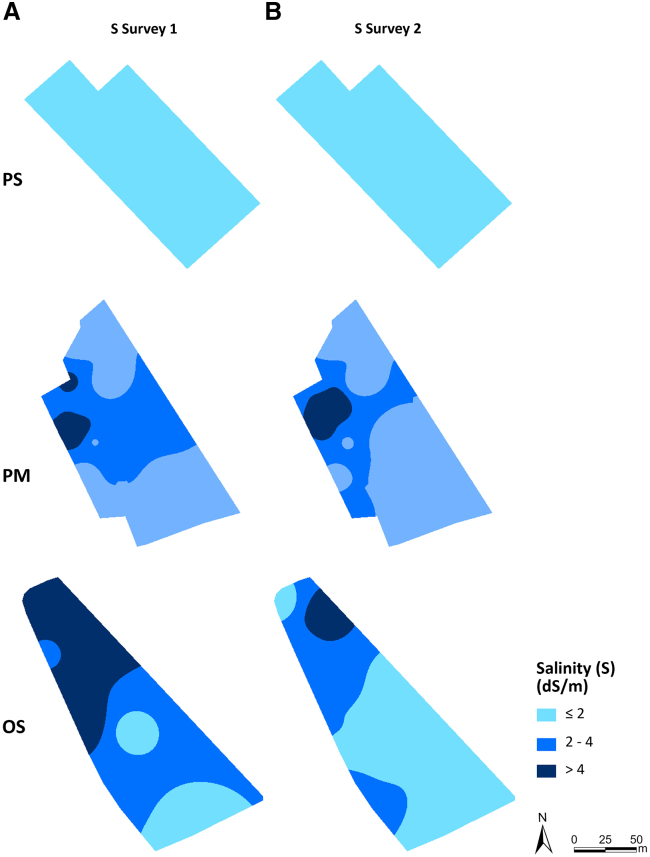
Salinity levels in the experimental fields (A) S1 – first survey on 12 July 2022, (B) S2 – second survey on 9 August 2022 PS- Polesinello soybean, PM Polesinello maize, OS- Oca Marina soybean.

In contrast to the soybean field, the maize field in the Polesinello locality exhibits higher salinity levels, along with noticeable shifts in saline areas between the first and second surveys (Table 2). Furthermore, observing Figure 2, the detrimental impact of high salinity levels becomes apparent, as evidenced by the lack of vegetation cover in the area with the highest salinity levels (as depicted in Figure 3). While some minor improvements are noticeable during the second survey, large portions of vegetation continue to exhibit signs of distress, as illustrated in Table 3.

**Table 2:** The area of salinity levels measured during the first and the second survey in the Polesinello maize field

Salinity (dS/m)	First survey (m^2^)	Second survey (m^2^)
≤2	10766.25	13309.5
2–4	8151.5	5103.75
˃ 4	982	1485

**Table 3:** The area of vegetation health levels measured during the first and the second survey and the change in vegetation health between the two surveys in the Polesinello maize field

Vegetation health	First survey (m^2^)	Second survey (m^2^)	Health change	Area (m^2^)
No vegetation	7350.62	3921.55	Reduction	3710.17
Suffering vegetation	2191.6	4655.85	No change	11522.34
Good vegetation	12312.01	11307.09	Improvement	4559.22

Figure 3 depicts the presence of various salinity levels, notably extended areas with high salinity levels, within the soybean field at the Oca Marina locality. Detailed information regarding the area occupied by each salinity level and the corresponding changes can be found in Table 4. Unlike in other fields, there were little to no signs of improvement in vegetation health between the first and second survey (Figure 2), possibly due to prolonged exposure to high salinity levels in this particular field (Figure 3). Further details are provided in Table 5.

**Table 4:** The area of salinity levels measured during the first and the second survey in the Oca Marina soybean field

Salinity (dS/m)	First survey (m^2^)	Second survey (m^2^)
≤2	4821.75	14227.25
2–4	9869.25	6610.75
˃ 4	7495.5	1347.5

**Table 5:** The area of vegetation health levels measured during the first and the second survey and the change in vegetation fitness between the two surveys in the Oca Marina soybean field

Vegetation health	First survey (m^2^)	Second survey (m^2^)	Health change	Area (m^2^)
No vegetation	2898.8	5746.1	Reduction	11338.26
Suffering vegetation	5733.63	11782.71	No change	10518.77
Good vegetation	13538.25	4641.88	Improvement	313.64

### Weed communities and their dynamics

**Figure 4:**
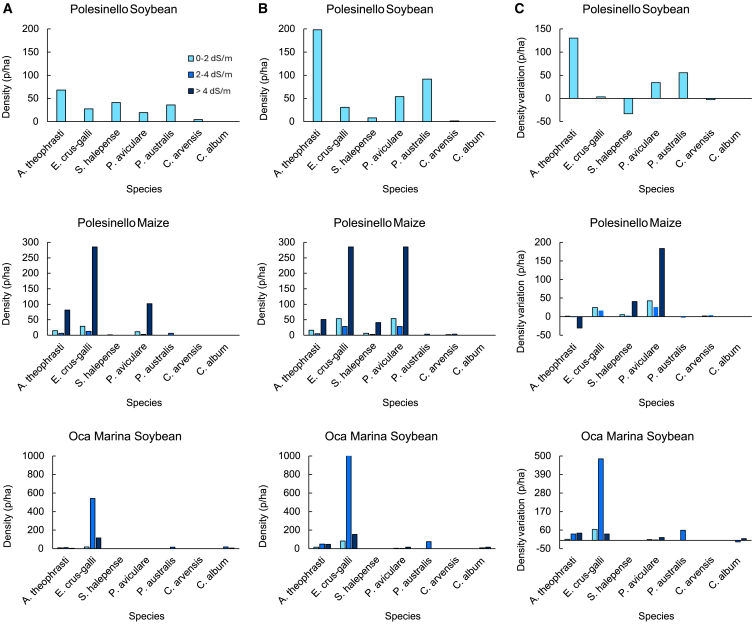
Weed species density in experimental fields (A) on 12 July 2022, first survey; (B) on 9 August 2022, second survey; (C) Density variation between the first and the second survey.

Despite their close proximity, notable differences were observed in the weed communities among the three fields studied. Moreover, distinct variations in weed species abundance and densities were evident (Column C), along with changes between the first (Column A) and second (Column B) survey periods (Figure 4).

As previously mentioned, both the density and species richness varied among the fields. However, the data in Figure 4 suggests that fields with lower salinity levels tend to exhibit higher species richness. Interestingly, the density of species does not consistently follow this pattern.

## Discussion

### Crop resilience under stress conditions

**Figure 5:**
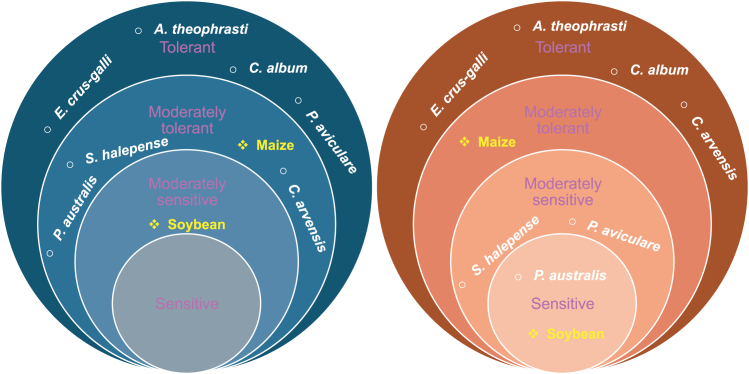
Salinity and drought tolerance of the crop and weed species present at the experimental sites

Although crops seem to struggle with salinity stress and drought, weeds, on the other hand, show much higher resilience (Figure 5).

Weeds are known for their ability to adapt to different environments and stress conditions, including high salinity, elevated temperatures, and drought.^67^^,^^68^^,^^69^ Indeed, the results obtained in this study clearly indicate that the weed species present at the study site were able to thrive, while crops were seriously struggling. The negative effects of varying salinity levels on major crops are well-documented.^4^^,^^70^^,^^71^^,^^72^ In this study, these negative effects on crops were particularly evident, especially in the case of soybean. This aligns with the literature, as soybean is usually defined as very susceptible to salinity stress, which can cause various damages such as high osmotic stress, water loss, homeostasis, and ion imbalances. Morphologically, salt-stressed soybean plants exhibit leaf chlorosis, necrosis, and scorching.^73^^,^^74^^,^^75^ At the same time, soybean is also a water-intensive crop and is very susceptible to drought conditions.^76^^,^^77^ On the other hand, maize is usually described as more tolerant to salinity stress, but when exposed to high salinity stress for extensive periods, the plants tend to reduce their biomass or even perish in extreme cases.^78^^,^^79^^,^^80^ Maize is also more tolerant to drought conditions, yet both the plant biomass and the yield will be seriously compromised if drought conditions persist,^81^^,^^82^ therefore it can be defined as a moderately drought-tolerant crop. A recent study by Nikolić et al.^83^ exposed soybean and maize to high salinity levels at varying temperatures, which clearly showed maize’s superiority in resisting salinity stress, consistent with our results (Figure 2).

### Weed adaptability to adverse conditions

The difference between the tolerance of crops and weed species to stress conditions can best be interpreted from the results presented in Figures 4 and 2. In Figure 4 it can be observed that the density of certain species, such as *Abutilon theophrasti* and *Echinochloa crus-galli*, in some fields, doubled from the first to the second survey. Plants of *A. theophrasti* were found in all three fields and at all three salinity levels. The most interesting result is the high density of this species in areas with extremely high salinity, where crops have completely perished (Figure 2), demonstrating this species’ high tolerance to adverse conditions and pronounced adaptation potential. There is evidence that this species is tolerant to both high salinity and drought conditions,^84^^,^^85^ which aligns with our results. The only example of *A. theophrasti* density reduction was seen in the maize field which, as stated above, is a relatively saline tolerant crop which allowed for better competition with this weed species.

According to Schmidt et al.^86^ and Karkanis et al.,^84^ *A. theophrasti* is capable of making different physiological and morphological changes, including shortening its life cycle, in order to produce seeds and disseminate, which seems to be a winning tactic in these conditions. *E. crus-galli* was another species present in all three fields and at each salinity level. Interestingly, this species had higher density at higher than at lower salinity levels, reaching 153 p/ha at high and 1023 p/ha at medium salinity levels, while these values were much lower at lower salinity levels, with a maximum of 82.96 p/ha. This species was also found to be tolerant to drought and salinity,^83^^,^^85^ which is consistent with the findings of this work. An additional factor contributing to this fast and massive expansion of *E. crus-galli* was probably the disappearance of crops, which significantly reduced competition for space,^87^ allowing this species to expand undisturbed.

An interesting behavior was also observed for *Sorghum halepense* and *Polygonum aviculare*, whose density increased or the species appeared in some fields from the first to the second survey, even in high salinity areas. These two species are described as tolerant to salinity but less tolerant to drought stress.^88^^,^^89^ This would explain the results obtained, as the probable cause of the increase in the number of these species was the rainfall event that occurred between the first and the second survey. However, some density reduction can also be noted in the Polesinello soybean field. The already fully developed plants likely succumbed to the drought stress, while the younger more vigorous ones survived and the new ones appeared after the rainfall events. Another similar example is *Phragmites australis*, whose numbers also increased in the second survey. This plant is described as susceptible to drought stress but tolerant to diverse salinity stress levels.^90^^,^^91^ Several studies have also highlighted significant variability in salinity tolerance among different populations of this species and across its various growth stages, with the germination stage being the most susceptible. Furthermore, research indicates that populations growing in saline environments exhibit greater salinity tolerance compared to those from non-saline environments.^91^^,^^92^^,^^93^This is consistent with the results obtained, as there were no plants of this species found in areas with high salinity levels, considering that this area does not usually experience rises in salinity, it can be assumed that the *P. australis* population found at the experimental site hasn’t yet developed high salinity tolerance.

*Chenopodium album* and *Convolvulus arvensis* are described as tolerant to both drought and salinity stress.^83^^,^^94^^,^^95^^,^^96^ In fact, *C. album* was present in high salinity areas in the Oca Marina soybean field during the first survey, which occurred during the drought period. Overall, the results indicate that in the case of salinity and drought stress, weeds will have the upper hand over crops.

### Environmental factors influencing salinity

All things considered, it is worth mentioning that salinization problems in coastal agricultural areas are well-documented.^49^ However, this level of damage was previously unreported in the Po River Delta; therefore, much of the damage to agricultural production witnessed can be attributed to the unexpected climatic phenomena that allowed and enhanced the soil salinization. Another critical point is the position of the fields, as higher salinity levels were measured in fields closer to the coast, as seen in the field in the Oca Marina locality. The vicinity of the sea and irrigation canals likely contributed to the rise of salinity, as conditions were favorable for saline water intrusion.^97^ Soil salinization can be greatly affected by the depth of the groundwater, where shallow water due to evaporation can seriously increase soil salinity levels.^98^ Considering that the study area is characterized by a shallow water table, saline water intrusion and the rise of underground waters, coupled with prolonged exposure to high temperatures, were the probable cause of an increase in soil salinity.^99^^,^^100^ Also, the well-known association between climate change, especially drought, and the rise of salinity can help us explain this phenomenon,^101^^,^^102^ considering that the meteorological data (Figure 1) clearly indicate prolonged drought conditions before and during the first survey, which likely contributed to the rise in salinity levels of the studied fields.

### Implications for agriculture and future research

Although this study focused on the Po River Delta, the results may be applicable to other similar areas in Italy and across Europe.^103^^,^^104^^,^^105^ These findings could help inform stakeholders and decision-makers about the often-overlooked but highly significant biotic stresses, such as weeds, which can thrive in hostile environments like high salinity and drought conditions. The combination of these biotic and abiotic stresses may have an even greater impact on agricultural production, particularly in coastal regions.

### Conclusions

Salinity and drought-related problems in agriculture are becoming increasingly important with the changing climate. Although the primary objective of plant production research is often focused on crop species, it is crucial not to neglect other organisms, such as weeds, that might become even more formidable adversaries under adverse conditions, as the results of this study suggest. The Po River Delta, as one of the regions most affected by salinization in northern Italy, provides a critical example of how such environmental stressors can influence agricultural ecosystems. The results of this study represent a significant advancement in understanding the impact of drought and salinity on agriculture in coastal areas, offering a unique perspective by assessing additional threats to crop production beyond traditional crop assessment methods. Moreover, it establishes a foundation for future studies by exploring appropriate methodologies for similar investigations. Additionally, our findings shed light on the potential effects of climate change on weed populations and their interaction with crops in vulnerable agricultural regions, an area that remains significantly understudied. This study underscores the remarkable competitiveness and adaptability of weeds compared to crops in the face of adverse conditions, such as the combination of drought and high salinity. The evidence presented here suggests that even under severe environmental stressors, weeds can complete their life cycles and propagate, potentially leading to long-term consequences for plant production. This resilience raises concerns about the accumulation of weed seeds in the seed bank, especially if fields remain abandoned due to economic reasons.

Furthermore, the rapid adaptability of weed species implies that future generations may carry enhanced genetic information for salinity and drought tolerance, posing an even greater challenge to crop cultivation in the future. Addressing these issues will require concerted efforts in agricultural research and management strategies to mitigate the impact of weeds on crop productivity and ensure sustainable agriculture in coastal regions such as the Po River Delta.

### Limitations of the study

Some of the limitations of this study should be acknowledged. The research was conducted in a single region, the Po River Delta, which could be considered a limitation for the broader applicability of the findings to other regions with different environmental or agricultural conditions. To reduce this limitation, three distinct fields with varying levels of soil salinity, crop cover, and weed flora were selected to enhance data robustness; also, the surveys were performed twice with also varying drought conditions. Although extending the study to other regions could provide broader applicability of the results, the variability introduced with the experimental design used significantly improves the robustness of the data and the projection of the results obtained to other similar regions. Additionally, data collection was limited to a single season, which restricts the ability to observe seasonal variations in weed communities and salinity levels. However, this season was particularly extreme and impactful with weather conditions difficult to replicate, offering valuable insights into how weeds might respond to harsh environmental conditions. Future studies could benefit from multi-season monitoring to better understand long-term trends. Finally, while UAVs with visible and multispectral sensors were used, optimizing drone flight parameters, such as altitude and sensor resolution, and improving weed species recognition algorithms could enhance the precision and efficiency of future studies.

## Resource availability

### Lead contact

Further information and resources requests should be directed to the lead contact, Paolo Tarolli (paolo.tarolli@unipd.it).

### Materials availability

No new unique materials, data or reagents were generated in this study.

### Data and code availability

• Data: the data reported in this paper are available within the main text or supplemental information and will be shared by the lead contact upon reasonable request.
• Code: this paper does not report original code.
• Other items: Any additional information required to reanalyze the data reported in this paper will be shared by the lead contact upon reasonable request.

## Acknowledgments

This study was carried out within the Agritech National Research Center supported by the European Union Next-GenerationEU (PIANO NAZIONALE DI RIPRESA E RESILIENZA (PNRR) – MISSIONE 4 COMPONENTE 2, INVESTIMENTO 1.4 – D.D. 1032 17/06/2022, CN00000022). The manuscript reflects only the authors’ views and opinions; neither the European Union nor the European Commission can be considered responsible for them.

## Author contributions

Conceptualization, N.N., E.S., P.T., R.M., and S.C.; methodology, N.N., E.S., P.T., R.M., and S.C.; investigation, N.N., E.S., and S.C.; writing – original draft, N.N.; writing – review and editing, E.S., P.T., R.M., and S.C.; funding acquisition, R.M.; resources, N.N., E.S., P.T., R.M., and S.C.; supervision, P.T. and R.M.

## Declaration of interests

We declare that P.T. is an advisory board member of the iScience journal, and the guest editor of the Special Issue entitled “Salinization of Soils in Agriculture: Threats, Monitoring and Mitigation”, and that he was not involved in the peer-review process of this paper.

## STAR★Methods

### Key resources table

**Table:** 

REAGENT or RESOURCE	SOURCE	IDENTIFIER
**Software and algorithms**		

ArcGIS Pro©	ESRI	https://www.esri.com/en-us/arcgis/products/arcgis-pro/overview

### Experimental model and study participant details

The experiments were conducted in the fields of soybean (*Glycine max*) and maize (*Zea mays*), in the Po River Delta region. The study focused on the naturally occurring, spontaneous weed flora, which was not influenced by the authors during the trials. The weed flora observed in the field included the following plant species: *Polygonum aviculare* (L.); *Chenopodium album* (L.); *Phragmites australis* ((Cav.) Trin. ex Steud.); *Sorghum halepense* ((L.) Pers.); *Convolvulus arvensis* (L.); *Abutilon theophrasti* (Medik.) and *Echinochloa crus-galli* ((L.) P.Beauv.).

The study was conducted under natural field conditions, with no artificial manipulation of soil or climate variables. No institutional approval was required for this study, as it was conducted in an open-field agricultural setting without direct intervention on plant populations.

### Method details

#### Study site

Experiments were conducted at Porto Tolle (Italy; Rovigo province), situated in the Po River delta, in two different localities: Polesinello and Oca Marina. The surveys were conducted over three fields, two with soybean (*Glycine max*), at the Polesinello and Oca Marina localities, and one with maize (*Zea mays*) located at Oca Marina locality (Figure 6). Soils of the experimental sites have low profile differentiation and sandy grain size, deep, coarse-textured.^106^ They are classified as Calcaric Arenosols by the^107^ and as Typic Ustipsamments, mixed, mesic by the Soil Survey Staff.^108^

**Figure:**
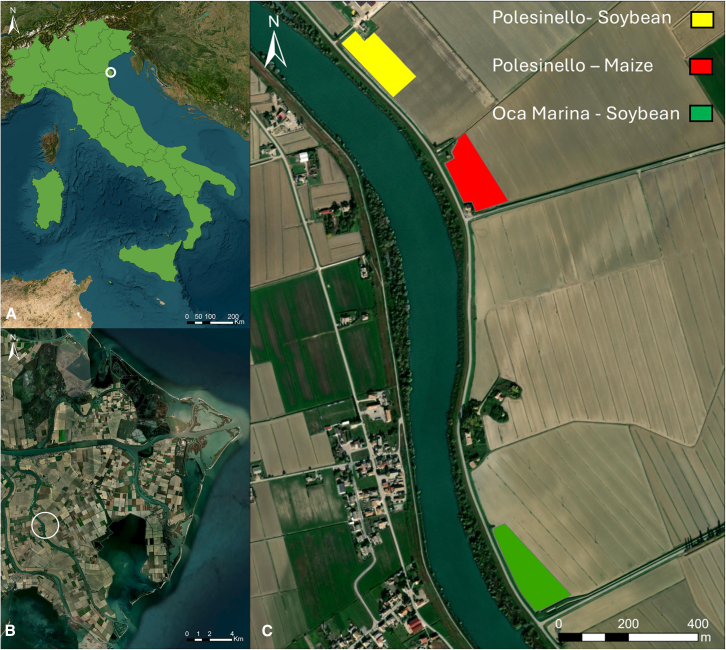
Experimental site (A) Position of the Po river delta within Italy. (B) The experimental area. (C) Position of the fields surveyed.

#### Surveys and weed mapping

Each field was surveyed twice during 2022, the first survey was conducted on 12^th^ July and the second one on 9^th^ August. The surveys utilized the Structure from Motion (SfM) technique with two UAVs: one for RGB survey and the other for multispectral purposes. A DJI Matrice 210 v2 with a Zenmuse X4S RGB camera (DJI Sciences and Technologies Ltd) was used for weed mapping, while a DJI Phantom 4 Multispectral (DJI Sciences and Technologies Ltd) was used for vegetation health assessment. Each flight was conducted at an altitude of 30 m above sea level (a.s.l.). The ground sample distance (GSD) was 0.0085 m for the DJI Matrice 210 v2 and 0.016 m for the DJI Phantom 4 Multispectral. Flights were performed around noon under predominantly clear skies to minimize shadow effects in the images. The surveys included some Ground Control Ponts (GCP) and 1/3 of them were used as Check Points (CP; Figure 7), measured with topographic Global Navigation Satellite System (GNSS; Geomax Zenith40; x,y,z accuracy: 0.02 – 0.03 m).

**Figure:**
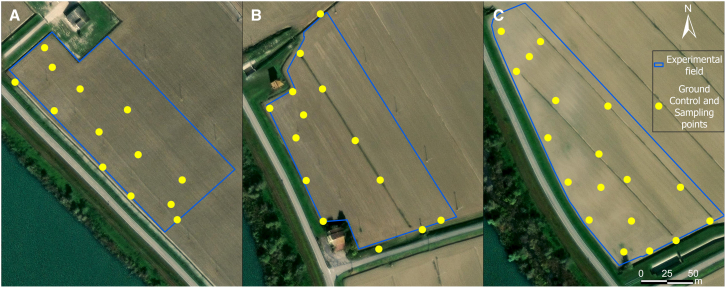
Ground control points and soil sample locations Example of the position of Ground Control Points (GCP) and soil samples (collected in the same location of GCPs) in each of the experimental fields: (A) 13 GCP and 13 soil samples in Polesinello study area cultivated with soybean; (B) 14 GCP and 14 soil samples “Polesinello” study area cultivated with maize; (C) 18 GCP and 18 soil samples Oca Marina study area cultivated with soybean

The outputs were high-resolution orthomosaics (2 cm per pixel), enabling accurate photo interpretation and weed species identification. Weed species presence and distribution were mapped, as shown in Figure 8, by creating polygons for different species, using Create Features function of ESRI ArcGIS Pro© software (v3.1.2).

**Figure:**
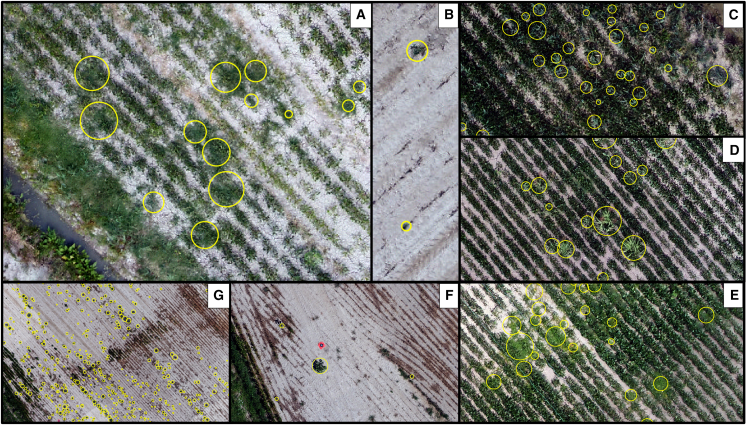
Weed species identification in the study area Example of different weed species identification: (A) *Polygonum aviculare*; (B) *Chenopodium album*; (C) *Phragmites australis*; (D) *Sorghum halepense*; (E) *Convolvulus arvensis*; (F) *Abutilon theophrasti*; (G) *Echinochloa crus-galli*

Precipitation and temperature data for summer 2022 were collected from the meteorological station of the Regional Agency for Environmental Prevention and Protection of Veneto (ARPAV) located nearest to the experimental fields.

#### Soil salinity assessment

During both UAV surveys, different soil samples were collected from all the experimental fields, following a randomized design of the GCP (Figure 7). The salinity level of soil samples has been determined in the laboratory following the protocol proposed by.^109^ Soil solutions were made using distilled water with a 1:2.5 weight/weight proportion. Prepared solutions were left in agitation for 24 h using Universal Table Shaker 70 (Instruments Lab Control s.b.c., Reggio Emilia, Italy). 24 h elapsed, the electrical conductivity of the samples was measured using XS Instruments COND 80 electrical conductivity meter (Giorgio Bormac s.r.l, Carpi, Italy) at a sensitivity of 1 μS. Salinity values obtained were then spatialized in the study areas, realizing salinity maps through the spatial interpolation tools of the GIS software. Following this procedure, the salinity of the experimental fields was classified into three classes: ≤ 2 dS/m low salinity, 2 – 4 dS/m medium salinity and ˃ 4 dS/m high salinity (Figure 3).

#### Vegetation health assessment

The data acquired during the first and the second survey using the multispectral camera were used to calculate the Normalized Difference Vegetation Index (NDVI) values of the vegetation cover present in the fields. The choice of this vegetation index was based on extensive literature reporting NDVI as ideal for estimating vegetation cover and plant health.^110^^,^^111^^,^^112^^,^^113^ Based on the regions of interest (ROI) for each orthophoto, the NDVI values were reclassified into several categories.^114^^,^^115^^,^^116^^,^^117^ Based on the available data, it was decided to create three classes of Vegetation Health (VH) to represent the absence of vegetation, suffering vegetation and vegetation in a good state. This process was conducted separately for each field by selecting distinct parts of the field representing vegetation in good condition, suffering vegetation, and barren land. Pixel values from these reference areas were used to define the thresholds for each vegetation health category. Specifically, areas with healthy vegetation were used to determine the pixel value range for the "Good vegetation" category, while areas with visibly stressed vegetation and barren land informed the "Suffering vegetation" and "No vegetation" categories, respectively.

Considering the differences in vegetation types and field conditions among the experimental fields, the thresholds varied for each field. For the Polesinello soybean field, the NDVI values were classified as follows: 0–0.4 for "No vegetation," 0.41–0.7 for "Suffering vegetation," and 0.71–1.0 for "Good vegetation." For the Polesinello maize field, the classifications were: 0–0.09 for "No vegetation," 0.091–0.1 for "Suffering vegetation," and 0.11–0.92 for "Good vegetation." Finally, for the Oca Marina soybean field, the NDVI thresholds were: 0–0.3 for "No vegetation," 0.31–0.6 for "Suffering vegetation," and 0.61–0.98 for "Good vegetation."The changes in vegetation levels between the first and the second survey were assessed by subtracting the two reclassified rasters (i.e., Difference of VH - DoVH) using the Raster Calculator function.

#### Determining weed species distribution

After creating the maps with different levels of salinity and weed species, it was possible to determine the number of plants for each weed species in the areas corresponding to a specific salinity level. This was accomplished by overlaying the salinity maps with that of different weed species. This approach established a spatial relationship between the two attributes of interest, salinity levels and different plant species. By employing this method, it was possible to extract the number of plants of different weed species that were growing at different salinity levels. This was performed by using the Spatial Join feature of the Analysis tools, which offers the possibility to join different attributes that share the same spatial distribution. This procedure was repeated for each weed species, providing the exact number of polygons, representing weed plants within the area of each salinity level.

Considering the area and the number of plants of different weed species, it was possible to represent the distribution of each weed species at each salinity level as a number of plants per hectare (p/ha). Considering that, due to weather conditions, salinity levels changed between surveys, it was decided to use the areas determined for salinity levels during the first survey, also for determining the weed species distribution for the second survey. This decision was made in order to always have the same reference areas, considering that the plants were exposed to those salinity levels during the germination and early growth stage (phenological phases crucial for plant survival).
